# Connecting the Dots: Linking Environmental Justice Indicators to Daily Dose Model Estimates

**DOI:** 10.3390/ijerph14010024

**Published:** 2016-12-28

**Authors:** Hongtai Huang, Timothy M. Barzyk

**Affiliations:** 1 Oak Ridge Institute for Science and Education (ORISE) at U.S. Environmental Protection Agency, National Exposure Research Laboratory, 109 T.W. Alexander Drive, Research Triangle Park, NC 27711, USA; 2 U.S. Environmental Protection Agency, National Exposure Research Laboratory, Research Triangle Park, 109 T.W. Alexander Drive, Research Triangle Park, NC 27711, USA; Barzyk.Timothy@epa.gov

**Keywords:** environmental justice, risk assessment, multiple stressors, dose estimates

## Abstract

Many different quantitative techniques have been developed to either assess Environmental Justice (EJ) issues or estimate exposure and dose for risk assessment. However, very few approaches have been applied to link EJ factors to exposure dose estimate and identify potential impacts of EJ factors on dose-related variables. The purpose of this study is to identify quantitative approaches that incorporate conventional risk assessment (RA) dose modeling and cumulative risk assessment (CRA) considerations of disproportionate environmental exposure. We apply the Average Daily Dose (ADD) model, which has been commonly used in RA, to better understand impacts of EJ indicators upon exposure dose estimates and dose-related variables, termed the Environmental-Justice-Average-Daily-Dose (EJ-ADD) approach. On the U.S. nationwide census tract-level, we defined and quantified two EJ indicators (poverty and race/ethnicity) using an EJ scoring method to examine their relation to census tract-level multi-chemical exposure dose estimates. Pollutant doses for each tract were calculated using the ADD model, and EJ scores were assigned to each tract based on poverty- or race-related population percentages. Single- and multiple-chemical ADD values were matched to the tract-level EJ scores to analyze disproportionate dose relationships and contributing EJ factors. We found that when both EJ indicators were examined simultaneously, ADD for all pollutants generally increased with larger EJ scores. To demonstrate the utility of using EJ-ADD on the local scale, we approximated ADD levels of lead via soil/dust ingestion for simulated communities with different EJ-related scenarios. The local-level simulation indicates a substantial difference in exposure-dose levels between wealthy and EJ communities. The application of the EJ-ADD approach can link EJ factors to exposure dose estimate and identify potential EJ impacts on dose-related variables.

## 1. Introduction

Since the early 1990s, Environmental Justice (EJ) advocates have expressed concern that using traditional risk assessment (RA) as a regulatory tool was inadequate in accounting for unusual exposures and susceptibilities, and could potentially cause environmental inequity [1,2]. A more comprehensive risk assessment process that integrates demographic data and fairly assesses risk distribution was recommended [3]. In 2003, the U.S. Environmental Protection Agency (EPA) developed a framework for cumulative risk assessment (CRA) which was defined as a procedural and analytical tool intended to characterize and possibly quantify the combined risks to human health or the environment from multiple agents or stressors [4,5]. Examples of CRA application include providing guidance for risk assessment in Superfund sites, combining ecological risks, and informing regulations and policies regarding pesticide controls and pollutants emission [5]. In addition to assessing cumulative risks from exposure to multiple chemical stressors or mixtures [6,7,8], CRA also considers other non-chemical factors [9,10,11,12,13]. For example, smoking will elevate the risks of having lung cancer associated with radon exposure [14,15]; toluene and noise together will induce higher levels of hearing loss [16]; children exposed to violence will have higher risks of developing asthma in the presence of air pollution [17]. This unique aspect of integrating socio-demographic information in CRA makes it different from conventional RA and allows it to address EJ issues as potential modifiers to exposure and dose-response.

On one hand, several quantitative tools have been established to help assess cumulative impacts and address EJ issues [18]. For example, the Office of Environmental Health Hazard Assessment (OEHHA), on behalf of California Environmental Protection Agency (Cal/EPA), developed a screening tool, CalEnviroScreen [19], for developing cumulative impact scores for communities as a response to scientific findings of EJ and associated health impacts, especially with respect to relevant local environmental laws and regulations [20]. Another example involves the EPA’s Environmental Justice Mapping and Screening Tool (EJSCREEN), which was designed to map environmental and demographic indicators at a detailed geographic level and offer screening information to support EJ research and policy development [21]. However, these tools do not provide direct estimates of exposure dose levels or the modifying impacts of EJ susceptibility/vulnerability factors on them. On the other hand, various exposure modeling techniques have been constructed to estimate environmental exposure doses. For examples, a personal delivered-dose model was developed to assess the impact of tetrachloroethylene on breast cancer risk via drinking water exposure [22], and a mechanistic dosimetry model was constructed to describe how respirable particles move in human airways [23]. Very few of the existing quantitative tools integrate EJ information and their specific contributions to exposure dose estimates. Our research begins to draw those linkages.

This work proposes an application to use existing approaches that can estimate exposure dose and incorporate EJ factors that modify them, termed the Environmental-Justice-Average-Daily-Dose (EJ-ADD). The concepts of “exposure” and “dose” are closely related and were sometimes used interchangeably [24]. The term “exposure dose” in this paper refers specifically to dose that is related to “temporally-integrated exposure” [24]. Dose is defined as “the amount of agent that enters a target in a specified period of time after crossing a contact boundary” [25].

Two components involved in this approach are the Average Daily Dose (ADD) model and EJ indicators. The former is an existing quantitative approach commonly used in RA and the latter is a measure of EJ susceptibility/vulnerability factors often included in a CRA framework. Therefore, EJ-ADD also represents a connection between RA and CRA. The RA framework includes five steps: problem formulation, hazard identification, dose response assessment, exposure assessment, and risk characterization [26]. A very important tool for exposure assessment in RA is the ADD model that has four main variables: contaminant concentration, intake rate, exposure factor, and body weight [27].

EJ indicators are essentially data that emphasize particular aspects of environmental or communal conditions and trends that could differentially impact environment-health relationships [28,29,30], providing useful information to support further public decision making [13,31]. EJ indicators have been used as a tool to assess and quantify non-chemical factors [20,32,33], including health, economic, and social indicators related to vulnerability and susceptibility [31]. Vulnerability factors typically encompass socio-economic conditions such as poverty, racial/ethnic bias, or education [34,35]; while susceptibility relates to biological factors such as life stage, genetic predisposition, or pre-existing health conditions [3,36]. Several studies have identified combined impacts of EJ indicators and chemical stressors [37,38,39,40]. Considering poverty as a non-chemical EJ indicator, individuals or families with lower income were located more closely to commercial, industrial, or traffic areas [41]. Race/ethnicity is another important factor widely evaluated in EJ assessments. Certain racial/ethnic minorities, due to economic and political disadvantage, could be exposed to more toxic pollution than other groups currently and through time. For example, aggregated cancer risk burden across Harris County, Texas, was found to be associated with the proportion of Hispanic residents and those at social disadvantage [29]. The interrelation between poverty and race/ethnicity with respect to environmental exposures has also been explored [42,43,44].

In this study, we demonstrate the utility of the EJ-ADD approach at both the nationwide and local levels. First, we quantified EJ indicators (poverty and race/ethnicity measures) for census tracts across the United States and joined them to ADD estimates of chemicals and chemical mixtures, using American Community Survey (ACS) data, the 2005 National-Scale Air Toxics Assessment (NATA), and the EPA Exposure Factors Handbook [25]. Poverty and race/ethnicity are indicative of many factors that could influence various components of the ADD model, such as exposure frequency and duration (for exposure factor), emission source prevalence (concentration), and public health (intake rate and body weight), all of which are concerns in EJ neighborhoods [9,21,41,45,46,47,48]. Second, based on a simulation of communities with different EJ-related scenarios, we estimated ADD levels for communities exposed to lead via soil/dust ingestion to show the utility of using EJ-ADD to estimate average daily dose with consideration of EJ indicators on a local scale.

## 2. Methods

### 2.1. Average Daily Dose Model

Our study used the ADD model/equation [27] to calculate the chemical average daily dose. ADD takes the product of contaminant/chemical concentration, intake rate, and exposure factor, divided by average body weight. The intake rate refers to the inhalation rate of contaminated air or ingestion rate of contaminated soil/dust, and exposure factor relates to the time period of contact with the contaminant (exposure duration) divided by averaging time [25], essentially the proportion of time exposed to the contaminant.

### 2.2. Nationwide Tract-Level Analysis

The nationwide tract-level analysis contains three major steps. We first calculated ADD at each tract for various chemicals (see below). We then defined and quantified two EJ indicators: poverty and race/ethnicity. Lastly, we matched the chemical ADD levels to the EJ indictors for each tract and analyzed their relationships.

#### 2.2.1. Chemical Concentrations

We used 2005 NATA pollutant concentration data (http://www.epa.gov/airtoxics/nata2005/) for ambient air at the tract level for five chemicals (acetaldehyde; benzene; cyanide; toluene; 1,3-butadiene) and particulate matter (PM) components of diesel engine emissions, namely diesel PM. These pollutants were selected based on their potential environmental influences, health impacts, and their relevance to EJ issues in that these chemicals are closely related to vehicular traffic and industrial emissions. Regulations regarding vehicle emission limits were set for four types of pollutants, including hydrocarbon, carbon monoxide (CO), nitrogen oxides (NO_x_), and diesel PM (https://www.epa.gov/regulatory-information-topic/regulatory-information-topic-air#transport). However, CO and NO_x_ were not included in the list of Air Toxics in the 2005 NATA Assessment (https://www.epa.gov/sites/production/files/2015-10/documents/2005-nata-pollutants.pdf) and therefore, we did not include these two chemicals but chose diesel PM as the representative pollutant. Except for lead compounds, other criteria air pollutants such as ground-level ozone and sulfur dioxide (SO_2_) were not included in the list of Air Toxics either. The lead level in air has been reduced by 98% over the past 30 years due to regulations of removing lead from gasoline (https://www.epa.gov/lead-air-pollution/basic-information-about-lead-air-pollution#how), so lead was also not selected in this exercise. This method can easily be adapted to other pollutants, but these were chosen as representative of industrial and vehicular emissions, as well as for their potential for health impacts. Exhaustive analyses of all industrial pollutants are beyond the scope of this study. Eventually, we matched the six chosen pollutants with the above-mentioned demographic data based on tract labels.

To attempt a more current analysis, we matched the 2005 NATA data with 2009–2013 ACS data, and performed the same procedural analyses. The results were nearly identical in terms of trending patterns except for small perturbations for certain chemicals.

#### 2.2.2. Age, Body Weight, and Intake Rate

We obtained age data for census tracts from the ACS database and then calculated the average weighted age for each tract. Specifically, age by sex for each tract is available via the 2005–2009 ACS 5-Year Summary file of the U.S. Census Bureau. In total, we evaluated approximately 65,000 tracts, representing around 305,000,000 people across the U.S. Note that census tracts are geographic units useful for presenting information for areas with population sizes representative of communities and neighborhoods [49]. The population sizes of census tracts range between 1200 and 8000 people and the average is around 4000, but their spatial sizes differ significantly from each other based on settlement density (https://www.census.gov/geo/reference/gtc/gtc_ct.html).

The 2005–2009 ACS data published in 2010 are five-year estimates based on data collected over a 60-month period, as opposed to “point-in-time” estimates that characterize demographic features of an area for a specific date/time. Therefore, a 2005–2009 data point is neither the 2007 average estimate nor an average based on 60 monthly values, but an average estimate based on information collected “continuously nearly every day of the year” and aggregated over five years. Multiyear estimates are especially advantageous and reliable for analyzing small geographic areas with populations of less than 65,000, such as for a community [49].

The weighted average age was calculated by summing the products of the percentage of each age group and the median (or predefined value if there was no median) of the corresponding age interval. For example, if the age groups (0, 10), (10, 20), (20, 60), and (60, ∞) for males in a tract are 10%, 20%, 40%, and 30% respectively, then the weighted age for that particular tract is 5 × 10% + 15 × 20% + 40 × 40% + 90 × 30%. Note that 90 is a predefined value here. Next, we calculated the mean value of all the weighted ages across all tracts for both male and female. Lastly, we obtained the mean of the female and male average weighted ages that will be used for further analysis.

The EPA Exposure Factors Handbook provides recommended values for long-term inhalation exposure and body weight according to age groups ranging from birth (<1 month) to adult [25]. Based on average weighted age, we identified the corresponding average body weight and 95th percentile intake rates for each tract by referring to the Exposure Factors Handbook, particularly, “Table 6-1 Recommended Long-Term Exposure Values for Inhalation” and “Table 8-1 Recommended Values for Body Weight” (see Supplementary Materials Tables S1 and S2).

#### 2.2.3. Data Analysis

We performed analyses using statistical software R (version 3.2.1; R Core Team, Vienna, Austria). Analyses include the calculation of ADD for single chemicals and chemical mixtures, calculation of EJ race and poverty scores, and a nationwide tract-level evaluation of exposure dose estimates based on the EJ indicators.

• Single Chemical ADDs

We assessed exposures to each of the six pollutants individually by calculating ADD using chemical concentration, inhalation intake rate, and body weight. We assumed the exposure factor to be 1, the maximum value, which means that residents living in a census tract were exposed constantly to the ambient air concentrations of each pollutant. Arguably, EJ communities would incur a higher exposure factor due to their living, working, and going to school near pollution sources; the disproportionate nature of this variable is a potential subject for further study. To compare the changes in ADD levels across different pollutants, we normalized the average ADDs associated with each score category for each chemical. Specifically, we divided each ADD by the maximum ADD value across all score categories for each chemical, with the unit of results being a percentage.

• Multiple Chemical ADDs

To evaluate exposure to a chemical mixture, we calculated the mixture ADD levels for acetaldehyde, benzene, and 1,3-butadiene using the Index Chemical Equivalent Dose (ICED) formula [4]. We selected benzene as the index chemical, the ‘well-studied component of the chemical mixture’ [4]. When calculating the chemical mixture dose, the doses of acetaldehyde and 1,3-butadiene were scaled to doses of benzene by using the Relative Potency Factor (RPF), which is the potency of one chemical relative to that of another [50] and a generalized form of the toxicity equivalence factor (TEF) method [4,51]. In this study, we used estimated inhalation unit risk to represent the potency of each chemical. According to the EPA Integrated Risk Information System (IRIS) (http://www.epa.gov/iris), the quantitative estimate of carcinogenic risk from inhalation exposure for 1,3-butadiene, acetaldehyde, and benzene are 3 × 10^−5^ per µg/m^3^, 2.2 × 10^−6^ per µg/m^3^, and 2.2 × 10^−6^~7.8 × 10^−6^ per µg/m^3^, respectively. We used 5 × 10^−6^ per µg/m^3^ (the median) as the unit risk for benzene. Therefore, the RPF of 1,3-butadiene to benzene was 6, and that of acetaldehyde to benzene was 0.44. The total chemical ADD level was the sum of the doses of each chemical weighted by the RPF. For example, when we considered chemical mixture exposure for both benzene and 1,3-butadiene, we added the 1,3-butadiene ADD level multiplied by the RPF of 1,3-butadiene to benzene, or the adjusted 1,3-butadiene ADD level to the index chemical ADD level (benzene ADD).

• EJ Poverty and Race

In that poverty and race/ethnicity are the two main factors that are closely related to other EJ indicators such as proximity to contamination sites and pre-existing conditions, and emphasized in many EJ studies [3,29,33,41,52], we selected these two indicators in this study. The measurement of poverty can be multi-dimensional and involves the concept ‘deprivation’—a person is poor in certain dimension if s/he is deprived in that dimension [53]. Due to data availability, we utilized ratio of individual income to poverty level and race/ethnicity population percentage data at the tract level from the 2005–2009 ACS 5-Year Summary file to quantify the EJ indicators poverty and race/ethnicity. We calculated the percentage of residents whose ratio of income to poverty level in the past 12 months was below 1.5 for each tract; that is, the percentage population with an annual income that was less than one-and-a-half times the poverty level. A score ranging from 1 to 10 was assigned to each tract based on percentage. Specifically, the numeric range from 0 to 1 was binned evenly into 10 intervals, each associated with a score value from 1 to 10 (1 being the lowest levels of poverty and 10 representing the highest proportion of poor residents). This score is defined as the EJ poverty score. We also calculated, binned, and assigned a score (ranging from 1 to 10 as well) to the percentage of non-white population in each tract. This score is considered the EJ race/ethnicity score (referred to as “race score” in the remaining text) with 1 being lowest non-white population percentage (i.e., most residents are white) and 10 being highest non-white percentage. We calculated the number of tracts for each poverty and race score. Supplementary Materials Table S3 presents the number of tracts for each score combination of both EJ indicators. When the poverty score is greater than 7, the sample size was relatively small in comparison to other groups. These small sample sizes reflect the need for scrutiny outside the scope of this paper, as they could represent very specific conditions; therefore, these results are not reflected in the subsequent graphs and interpretations.

We matched EJ indicators to single- or multiple-chemical ADD values at the tract level, and then grouped census tracts based on their EJ indicator scores and score combinations. We then analyzed the relationship between EJ indicators and chemical ADDs. We first calculated the mean single-chemical ADDs associated with each score for each EJ indicator separately. We then calculated the mean single- or multiple-chemical ADDs associated with each unique combination of EJ indicator scores.

We used the function “persp3d” in the R package “rgl” [54] to generate the 3-dimesional (3-D) surface plots based on discrete matrix inputs. Details of how the surface was created can be found in the R document of package “rgl” (http://cran.R-project.Org/package=rgl). 3-D surface plots provide straightforward visualization and therefore can potentially improve communications between scientists and decision makers with less requirement of mathematical background.

### 2.3. Average Daily Dose Estimates of Local Scale Scenarios

Most analyses thus far were performed for all U.S. census tracts. However, the EJ-ADD approach can also be applied for specific communities or neighborhoods, which is sometimes more preferable and informative given that certain unusual exposure dose levels may not be easily discerned if one focuses on the average doses of a large geographic unit. Ideally, community-level data regarding body weights, intake rates, and exposure factor information should be available and can be used directly to estimate exposure dose using the ADD model. However, due to lack of data access, we estimated the ADD levels for communities with distinct environmental scenarios based on information from relevant peer-reviewed journal articles in order to demonstrate the utility of using EJ-ADD method to estimate average daily dose with consideration of EJ indicators on a local scale. Note that the inputs for the ADD model in the nationwide tract-level analysis were highly related to the variable age with less consideration of other EJ factors. In this exercise, we will demonstrate how EJ indicators can affect each parameter in the ADD model in a more direct fashion. We examined only lead exposure via soil/dust ingestion as an example here. The purpose of this exercise is to show the utility of using EJ-ADD on a smaller geographic unit, so exhaustively considering multiple routes of exposure to multiple pollutants is not the focus of this demonstration.

The first scenario is a wealthy community far away from any industrial or contamination sites. All the residents are living well above the poverty level and can access grocery stores easily. The next scenario involves an EJ community residing in areas severely affected by mining activities and we assume that the majority of the residents are living in poverty with limited access to grocery stores (i.e., living in “food desert”). The other two scenarios lie in between these two above-mentioned scenarios with closer proximity to the mining sites than the wealthy community but further than the EJ community.

The approximation of parameter values in the ADD model is as follows.

**C** (lead concentration in soil): We utilized results presented in Bergstrom et al. [55]. In particular, we first calculated the averaged geometric mean (GM) of lead concentration for each of the two reference soil samples weighted by the mass composition fraction and then assigned the rounded average of these two reference values (5 mg/kg) as the lead concentration in soil for the wealthy community. Similarly, we calculated the average of the weighted-averaged GM of the three samples collected downstream from the Bunker Hill Mining and Metallurgical Complex that was included in the EPA’s National Priorities List (NPL), and assigned the rounded value (5 × 10^3^ mg/kg) as the lead concentration for the EJ community. We assigned 50 mg/kg and 500 mg/kg as the lead level for the other two scenarios.

**BW** (body weight): We used 80 kg as the average population body weight for the wealthy community. A previous study showed that poverty rates were linked with obesity based on data from 3139 U.S. counties, and that the obesity rate of the wealthiest quintile (included 630 counties) is around 5% lower than that of the poorest quintile (encompassed 629 counties) [56]. Therefore, we assumed 84 kg (=80 kg × 1.05) as the BW value for the EJ community, and 82.4 kg (=80 kg × 1.03) and 83.2 kg (=80 kg × 1.04) for the other two communities respectively.

**IR** (intake rate, soil/dust ingestion rate in this case): Our estimation was based on the findings that increasing intake of foods was associated with behaviors leading to obesity [57]. In addition, psychosocial stress can also lead to eating disorder such as pica [58]. Therefore, we assumed the soil ingestion rate for residents in EJ communities was 70% higher than that of the wealthiest community where the residents’ soil ingestion rate was assumed to be 50 mg/day according to the EPA’s Exposure Factor Handbook. Furthermore, 60 mg/day (20% increase) and 75 mg/day (50% increase) were assumed to be the IR for those living in the two intermediate communities.

**EF** (exposure factor): We assumed the exposure factor to be 1 for the EJ community in that all the residents are constantly exposed to lead pollution on a daily basis. For the wealthiest community, it was assumed that exposure duration for most residents was 3 h/day and therefore, EF would be 0.125 (=3 h-day/24 h-day). Furthermore 0.25 and 0.5 were assigned to be the EF values for the other two scenarios.

We calculated the ADD levels for the four scenarios and compared them in order to evaluate how the exposure dose levels change when EJ indicators were considered for smaller geographic units.

## 3. Results

### 3.1. Nationwide Average Daily Dose Estimate

We evaluated ADD for the group of chemicals (normalized for graphing) for poverty and race/ethnicity separately, and then examined each chemical with respect to the combination of EJ indicators, as both 2- and 3-dimensional plots. Finally, we analyzed race/ethnicity and poverty with respect to a chemical mixture.

#### 3.1.1. Single EJ Indicator

**Figure 1:**
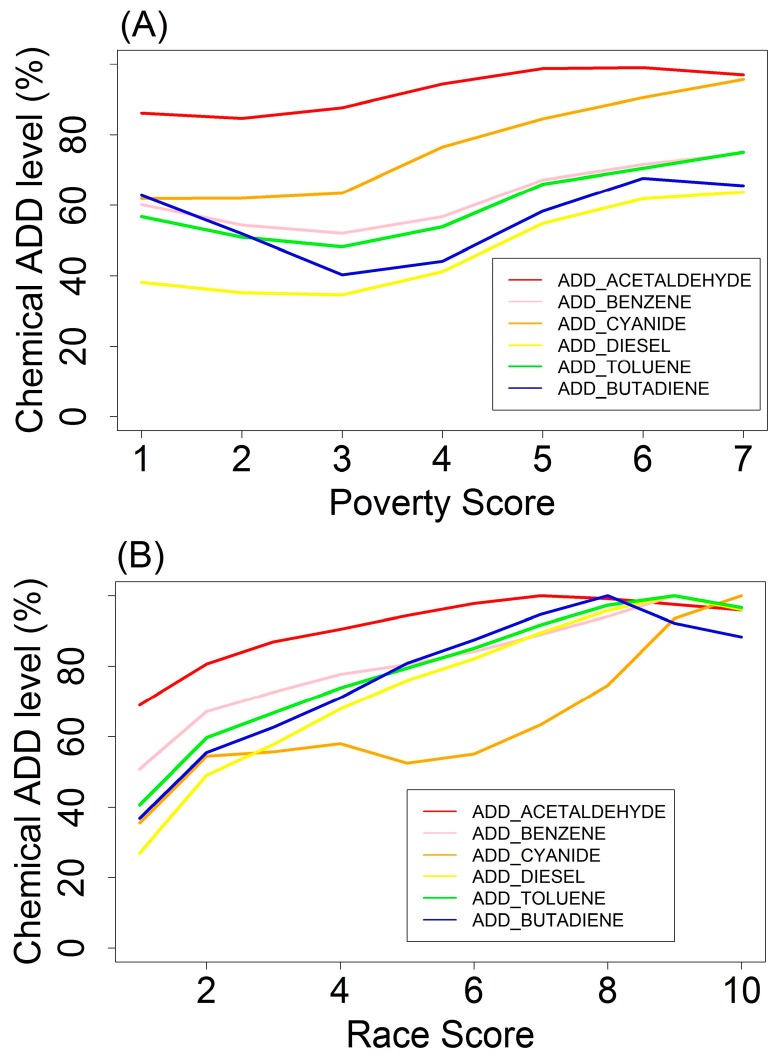
Single-chemical Average Daily Dose (ADD) level (%) associated with EJ scores. (**A**) Poverty Score; (**B**) Race Score.

In Figure 1, it can be seen that the ADD level for most pollutants increased as the poverty score increased from 2 to 7. The ADD level of cyanide had the highest increase (33.64%) and that of acetaldehyde the least (14.34%). 1,3-Butadiene showed relatively similar ADD levels at poverty scores of 1 and 2, as compared to higher poverty scores (6 and 7).

Figure 1 also shows the trend of chemical ADD with respect to increasing race score. The ADD level of most chemicals (except cyanide compound) increased gradually as race score became larger, reached the peak at race score of 8, and decreased slightly as race score increased from 8 to 10. The ADD level of diesel PM had the greatest increase (73.00%) and that of acetaldehyde the least (31.03%). Similar to chemical ADD levels associated with poverty score, aside from isolated perturbations, ADD displayed monotonically increasing trends as race score became higher.

#### 3.1.2. Two EJ Indicators (Multiple Chemical)

**Figure 2:**
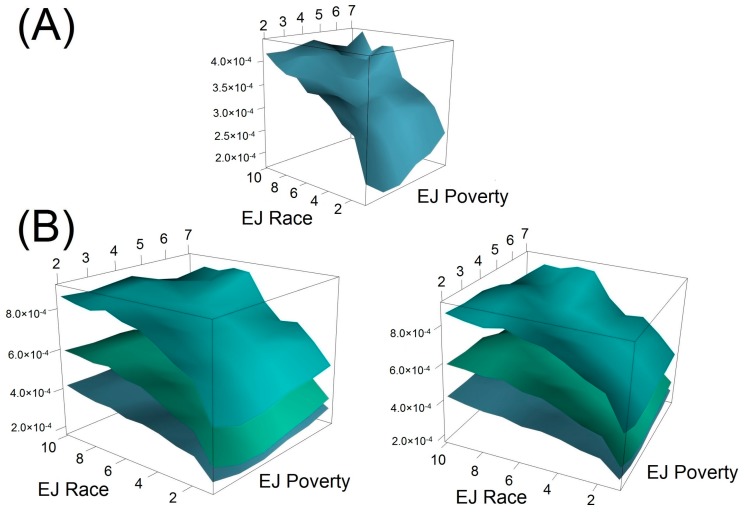
Two Environmental Justice (EJ) Indicators 3D Plot. (**A**) Single-chemical (benzene) ADD levels (mg-day/kg) associated with both poverty and race scores (color in sky blue); (**B**) Multiple-chemical ADD levels (mg-day/kg) associated with both poverty and race scores. These two graphs are snapshots of different perspectives of the same 3-D plot. The bottom layer (color in sky blue) represents the ADD level of benzene; the middle layer (color in aquamarine) represents the ADD level of benzene plus that of adjusted 1,3-butadiene; the first layer (color in cyan) represents the ADD level of benzene plus that of both adjusted 1,3-butadiene and acetaldehyde.

Figure 2 shows the 3D plot of benzene ADD with respect to both poverty and race/ethnicity, indicating a saddle-like surface to the results. As race score increased from 1 to 10, the benzene ADD level increased by more than 100% (from 2 × 10^−4^ mg-day/kg to more than 4 × 10^−4^ mg-day/kg). Comparing between EJ indicators, increasing poverty scores generally resulted in higher benzene ADD as well, but with smaller magnitude. When poverty and race scores were in their medians, there was a flatter surface (lower slope) for benzene ADD levels. A more detailed analysis on the suggested impacts of poverty and race/ethnicity on ADD are presented in the Discussion section below. Results of analysis for two EJ indicators with single chemical can be found in Supplementary Materials Figure S1.

Figure 2 also illustrates the 3D surface plots for three layers of chemical ADD: benzene, benzene plus adjusted 1,3-butadiene, and benzene plus adjusted 1,3-butadiene and adjusted acetaldehyde. All three layers of ADD followed almost the same pattern as the single chemical 3D plot. However, the perturbations in the ADD surface for multiple chemicals were larger than those for a single chemical (i.e., benzene).

### 3.2. Dose Estimates of Local Scale Scenarios

In Table 1, it can be found that the lead exposure dose level in the simulated EJ community is around 12,941 (=5.06/3.91 × 10^−4^) times more than that in the wealthiest community. The lead ADD of the two intermediate communities are also substantially higher than the most affluent neighborhood by 2 or 3 orders of magnitude. As the poverty level of the community increased, the BW increased as a denominator and can result in a decrease in ADD while holding other parameters constant. However, its change cannot outweigh that of other parameters. Therefore, in this simulated application, the ADD level of the poorer community is still tremendously higher than that of the wealthy one.

## 4. Discussion

In general, we found that each EJ indicator (poverty and race/ethnicity) was positively associated with higher chemical exposure dose estimates based on a nationwide tract-level evaluation. Racial/Ethnic minority and poor neighborhood combined were also associated with higher chemical exposure levels. The local-level simulation indicates a substantial difference in exposure-dose levels between wealthy and EJ communities.

### 4.1. EJ-ADD Approach

Different from applying other modeling techniques or tools, the application of the EJ-ADD approach can both estimate average daily dose and take into account environmental justice concerns at different geographic levels. In particular, it has the following advantages in addressing EJ issues. First, it can provide direct estimates of exposure dose level for EJ communities and vulnerable population. Both CalEnviroScreen and EJSCREEN are, in essence, cumulative impact mapping tools of which the outputs are used to inform public environmental decision making related to resources allocation and identification of EJ communities. EJ-ADD allows further quantification of exposure doses for the identified communities with EJ concerns. Second, this approach integrates both environmental exposure dose information and demographic data, which is a valuable advantage most exposure and dose modeling techniques tend to neglect. Although the personal delivered dose model [22] integrates individual behavior information such as consumption and bathing habits, other EJ indicators related to extrinsic vulnerability such as “food deserts” and poverty cannot be taken into account when estimating dose levels using the personal delivered dose model. Examination of EJ indicators that influence exposure can be conducive to targeting risk-reduction actions, such as an education outreach program, installing air filters in houses close to contamination sites, or an emission reduction campaign. Third, the EJ-ADD approach is not limited to estimating dose level for a particular route of exposure, as demonstrated by both the nationwide tract-level analysis (the media type was air) and the local-scale simulation exercise (the media type was soil/dust). It can also be applied to estimate exposure dose for other media such as water provided that all the input information is available. Fourth, partly derived from dose modeling techniques in conventional RA, EJ-ADD is complementary to conventional RA and can inform public decisions with consideration to EJ issues for which conventional RA alone cannot achieve.

### 4.2. Average Daily Dose Estimates for U.S. Census Tracts

We focused mainly on chemical exposures and EJ indicators at the census tract level, aggregated to represent areas with particular race/ethnicity and poverty conditions. It has been demonstrated that socio-economic status measured at the individual level compared to the area level could have independent impacts on individual health conditions [59]. Therefore, our results may not be consistent with other studies concentrating on individual-measured chemical exposure levels.

When we considered only one EJ indicator, we found that both EJ poverty and EJ race were positively associated with ADD exposure levels. This finding is consistent with results in other environmental justice literature [3,10,29,41,42,44,60,61,62], suggesting that the approach we adopted is reasonable and could be utilized for future studies.

If we evaluate both EJ indicators simultaneously (see Supplementary Materials Figure S1), we observe an increasing trend in chemical exposure levels as the percentage of non-white residents increased from 0% to 100% for most chemicals. Chemical exposures among the different poverty scores cannot be easily differentiated for race scores <6 (i.e., <60% non-white), after which, higher poverty scores tended to group at higher ADD levels, and lower poverty scores (i.e., wealthier tracts) grouped toward lower exposure levels. Poverty further aggravated the chemical exposure levels in conjunction with race/ethnicity, inducing higher chemical exposure levels for lower-income census tracts. In most cases, once race score reached 6 (≥50% non-white), poverty began to differentiate between chemical exposures more clearly, with lower poverty scores (higher percentage of low-income residents) achieving higher levels, and vice-versa. Exposure for race score <6 showed less differentiation (i.e., tighter grouping) of ADD across poverty scores, yet exposures still increased with race score. In all cases, ADD for non-white areas continued to increase with race score; for example, tracts with more than 80% non-white residents show at least a 300% increase in diesel PM exposure compared to those with less than 20% non-white residents. For five out of six pollutants (except for cyanide compounds), exposure levels slightly decreased as the non-white percentage increased from 80% to 100% (i.e., as race score increased from 9 to 10) across most poverty conditions, yet ADD levels were still greater than at the lower race scores (i.e., between 1 and 5).

The 3-dimensional surface for benzene (Figure 2) provides additional clues as to the interactions between race/ethnicity and poverty with respect to chemical ADD levels. As evidenced by the graph, there is a sharp increase in ADD as race score increases from 1 to 5, regardless of poverty, which actually shows no clear monotonic increase from higher- to lower-income tracts (i.e., from low to high poverty scores). However, within the middle of the surface, between poverty scores 4–5 and race scores 5–7, there exists a shallower region surrounded on three sides by sharp increases in slope (i.e., toward higher or lower poverty or toward higher race scores). This area is of particular interest because it represents an area of median characteristics, approximately 50% non-white and 50% low-income.

From this region, holding race score constant, as poverty score increases from 3 to 7, ADD also increases across all three different layers, which might be expected. However, as poverty score decreases from 3 to 2, ADD still slightly increases, suggesting that higher-income areas are not necessarily offering additional protections to these racially/ethnically mixed areas. Also from this region, very clearly, as race score increases from 1 to 8, then ADD also increases dramatically, regardless of whether poverty remains constant, increases, or decreases. In all cases, race/ethnicity is a clearer indicator of higher ADD exposure levels in this study, and economic advantage does not necessarily offer non-white populations additional protection from chemical exposures. Future work will include additional geospatial analyses in an attempt to distinguish circumstances that could account for this pattern (e.g., the geographic distribution of high-, medium-, and low-income areas with various proportions of non-white population); contributing factors could include urban, suburban, and rural conditions, or socio-political factors such as historical discrimination and the inertia of racial/ethnic segregation through time.

### 4.3. Average Daily Dose Estimates of Local Scale Scenarios

Without explicit statement regarding which EJ factors may potentially impact exposure dose estimates in the local-scale simulation, this exercise in fact involves four different EJ indicators when making assumptions and assigning values for the parameters in the ADD model, including proximity to NPL, pre-existing condition (e.g., obesity and soil pica), poverty, and food deserts. It highlights the merit of using EJ-ADD as a useful application to address EJ concerns which other exposure dose modeling tools tend to neglect.

Our simulated results indicate that the difference in exposure dose levels between wealthy and EJ communities can have multiple orders of magnitude when considering multiple EJ indicators simultaneously. The cumulative impacts of these EJ indicators can be reflected quantitatively via the application of EJ-ADD in a way that other techniques cannot achieve. In future analyses, if one provides a list of EJ indicators to be considered before exposure dose estimates, EJ-ADD may serve as a reasonable approach to start with.

### 4.4. Other EJ Indicators

We only considered percentage non-white and ratio of income to poverty explicitly as EJ indicators in the nationwide tract-level analysis, although the NATA tract-level pollutant concentrations and the ACS age information could also be viewed as EJ indicators such as those defined in previous studies [63]. However, additional EJ indicators can potentially be integrated using our approach.

Race/ethnicity and poverty are indicators of environmental injustice, and can be used to identify and characterize EJ areas and demonstrate (as shown here) their relation to chemical exposures. However, they are larger issues that, in and of themselves, do not explain the reasons that EJ populations are over-exposed (i.e., vulnerability), nor do they account for greater risk based on human dose-response (i.e., susceptibility). In order to fully deconstruct and investigate the negative impacts of environmental injustice, a further examination (at finer granularity than race/ethnicity and poverty) of the socio-economic and biological factors associated with them is warranted.

Supplementary Materials Table S4 presents a number of EJ-related variables that could potentially modify the likelihood or consequence of either exposure or response to chemical pollutants, and have thus been termed, exposure/response modifiers or ERMs. ERMs encapsulate susceptibility and vulnerability factors without requiring a distinction between them, since both could contribute to increased biological risk, such as food deserts (a vulnerability) being related to biological resiliency (a susceptibility).

### 4.5. Limitations

Four major limitations of this study are discussed in this section and can potentially be investigated in future analysis.

First, we used ADD instead of Lifetime Average Daily Dose (LADD) [27], in that we do not have sufficient exposure duration and frequency information to accurately assess LADD exposure factors on a national scale. We made the assumption of exposure factor being equal to 1 when calculating ADD. The influence of this assumption would be magnified if we used LADD as the basis to estimate chemical exposures. Provided we have enough temporal information for exposure factors at a community level, LADD can possibly be used interchangeably with the ADD model for exposure dose estimates.

Second, when considering multiple chemical exposures, the pattern of interactions between EJ indicators became more evident. In this work, we made the simplified assumption that different chemicals did not interact with each other in a chemical mixture, which allowed us to utilize the ICED formula. We are aware that such an assumption may not hold in different environmental settings. In addition, other potency measures may be used to calculate the relative potency factor for different chemicals. We understand that the unit risk value of benzene provided by IRIS is represented by a range and not a single value. However, due to the ubiquitous nature of benzene and to estimate the average benzene ADD level on a national scale, we had to make certain simplifications within a reasonable scope, and therefore chose to utilize the median value for inhalation unit risk. The main purpose of showing this chemical mixture result is to demonstrate the ability of employing this ADD-EJ approach to analyze interactions between EJ indicators and chemical mixtures, acknowledging that EJ communities are often host to multiple chemical agents simultaneously and periodically through time.

Third, given that majority of this work was based on national-scale data, the quantitative relationship regarding the interactions between race scores and poverty scores cannot be applied to a particular area or city, and only for descriptive purpose. Mathematically identifying the optimal balance between these two scores in terms of minimizing chemical exposures is beyond the scope of this paper but could certainly be explored in the future.

Fourth, in the local-scale exercise, we used simulated scenarios in cases where we did not have relevant local level data to estimate ADD levels for the communities with different characteristics. Ideally, the estimated ADD levels of these communities could be matched directly to EJ indicators similar to what we did for the nationwide tract-level analysis, thus exploring the corresponding relationships between ADD and EJ indicators.

## 5. Conclusions

The purpose of this study was to identify an approach that links EJ indicators to exposure dose estimates. First, we presented a nationwide tract-level analysis of ADD, organized by EJ scores for poverty and race/ethnicity. We found that these EJ indicators likely contribute to increased exposure dose, which is supported by the EJ literature. Then, we presented a simulation of communities, representative of a spectrum from affluent to EJ-impacted, to demonstrate the utility of EJ-ADD on a local scale.

By linking conventional RA methods, such as the ADD model, to exposure response modifiers typically found in CRA applications, such as proximity to NPL, EJ-ADD can evaluate the potential primary contributing factor of increased exposure and dose, in the hopes of developing more targeted solutions.

Finally, this work seeks to bridge the gap between conventional RA and broader CRA considerations and applications. This study has no intention to be separated from classical techniques of exposure dose modeling, but rather to draw more attention to EJ indicators that can influence modeling variables.

We conclude that the application of the EJ-ADD approach can link EJ factors to exposure dose estimate and identify potential impacts of EJ factors on dose-related variables.

## Figures and Tables

**Table 1:** Parameters and ADD Estimates for Four Different Scenarios. BW, body weight; IR, intake rate; C, lead concentration in soil; EF, exposure factor.

	Wealthiest Community	Middle Community 1	Middle Community 2	EJ Community
**BW (kg)**	80	82.4	83.2	84
**IR (mg/day)**	50	60	75	85
**C (mg/kg)**	5	50	5.00 × 10^2^	5.00 × 10^3^
**EF**	0.13	0.25	0.50	1
**ADD (mg/kg-day)**	3.91 × 10^−4^	9.10 × 10^−3^	0.23	5.06

## Supplementary Materials

The following are available online at www.mdpi.com/1660-4601/14/1/24/s1, Table S1. Exposure Factors Handbook (U.S. EPA 2011) (Table 6-1. Recommended Long-Term Exposure Values for Inhalation); Table S2. Exposure Factors Handbook (U.S. EPA 2011) (Table 8-1. Recommended Values for Body Weight); Table S3. Number of census tracts associated with EJ scores; Table S4. Other EJ-related Exposure/Response Modifiers; Figure S1. Single-chemical ADD levels (mg-day/kg) associated with both poverty and race scores.
