# Supplementary material for: Connecting the Dots: Linking Environmental Justice Indicators to Daily Dose Model Estimates
Source: Int J Environ Res Public Health. 2016 Dec 28;14(1):24. doi: 10.3390/ijerph14010024 (PMC5295275; doi:10.3390/ijerph14010024)
Supplement: Supplementary file 1 [file ijerph-14-00024-s001.pdf]

Supplemental Materials: Connecting the Dots: Linking Environmental Justice Indicators to Daily Dose Model Estimates

Hongtai Huang and Timothy M. Barzyk

Table S1. Exposure Factors Handbook [1] (Table 6-1 Recommended Long-Term Exposure Values for Inhalation).

Table 6-1. Recommended Long-Term Exposure Values for Inhalation (males and females combined)

Age Group[a]	Mean (m^3/day)	Sources Used for Means	95th Percentile[b] (m^3/day)	Sources Used for 95th Percentiles	Multiple Percentiles
Birth to <1 month	3.6	c	7.1	c	See Tables 6-4, 6-6 through 6-8, 6-10, 6-14, 6-15 (none available for Stifelman, 2007)
1 to <3 months	3.5	c, d	5.8	c, d	
3 to <6 months	4.1	c, d	6.1	c, d	
6 to <12 months	5.4	c, d	8.0	c, d	
Birth to <1 year	5.4	c, d, e, f	9.2	c, d, e	
1 to <2 years	8.0	c, d, e, f	12.8	c, d, e	
2 to <3 years	8.9	c, d, e, f	13.7	c, d, e	
3 to <6 years	10.1	c, d, e, f	13.8	c, d, e	
6 to <11 years	12.0	c, d, e, f	16.6	c, d, e	
11 to <16 years	15.2	c, d, e, f	21.9	c, d, e	
16 to <21 years	16.3	c, d, e, f	24.6	c, d, e	
21 to <31 years	15.7	d, e, f	21.3	d, e	
31 to <41 years	16.0	d, e, f	21.4	d, e	
41 to <51 years	16.0	d, e, f	21.2	d, e	
51 to <61 years	15.7	d, e, f	21.3	d, e	

a. When age groupings in the original reference did not match the U.S. EPA groupings used for this handbook, means from all age groupings in the original reference that overlapped U.S. EPA's age groupings by more than one year were averaged, weighted by the number of observations contributed from each age group. Similar calculations were performed for the 95th percentiles; b. Some 95th percentile values may be unrealistically high and not representative of the average person; c. Arcus-Arth A, Blaisdell RJ. 2007. Statistical distributions of daily breathing rates for narrow age groups of infants and children. Risk Analysis 27:97–110; d. Brochu P, Ducré-Robitaille J-F, Brodeur J. 2006. Physiological daily inhalation rates for free-living individuals aged 1 month to 96 years, using data from doubly labeled water measurements: A proposal for air quality criteria, standard calculations and health risk assessment. Human and Ecological Risk Assessment: An International Journal 12:675–701; e. U.S. EPA (Environmental Protection Agency) Washington D, EPA/600/R-06/129F, 2009. 2009. Metabolically derived human ventilation rates: A revised approach based upon oxygen consumption rates (final report) U.S. EPA, Washington, DC, EPA/600/R-06/129F. Available: http://ofmpub.epa.gov/eims/eimscomm.getfile?p_download_id=490080 (accessed on 2 February 2016); f. Stifelman M. 2007. Using doubly-labeled water measurements of human energy expenditure to estimate inhalation rates. The Science of the total environment 373:585–590.

Table S2. Exposure Factors Handbook (U.S. EPA 2011) (Table 8-1 Recommended Values for Body Weight).

Table 8-1. Recommended Values for Body Weight

Age Group	Mean (kg)	Multiple Percentiles	Source
Birth to <1 month	4.8	Tables 8-3 through 8-5	U.S. EPA analysis of NHANES, 1999–2006 data
1 to <3 months	5.9		
3 to <6 months	7.4		
6 to <11 months	9.2		
1 to <2 years	11.4		
2 to <3 years	13.8		
3 to <6 years	18.6		
6 to <11 years	31.8		
11 to <16 years	56.8		
16 to <21 years	71.6		
Adults	80.0		

NHANES, National Health and Nutrition Examination Survey.

Table S3. Number of census tracts associated with EJ scores.

			EJ Poverty									
	**Score**		**1**	**2**	**3**	**4**	**5**	**6**	**7**	**8**	**9**	**10**
		Total	13,146	17,703	13,955	9032	5429	3136	1674	779	252	76
**EJ Race/Ethnicity**	1	23,270	6503	8338	5286	2140	563	191	105	94	38	12
	2	12,825	3430	3873	2799	1480	615	280	186	104	53	5
	3	8200	1524	2147	1949	1293	667	336	148	99	31	6
	4	5572	668	1201	1272	1069	756	375	148	56	16	11
	5	4043	349	723	861	903	628	334	156	72	12	5
	6	2951	211	498	570	638	544	271	143	49	19	8
	7	2213	128	270	399	463	443	305	142	49	10	4
	8	1849	130	237	278	333	379	276	140	60	11	5
	9	1808	118	199	239	298	366	280	214	61	25	8
	10	2451	85	217	302	415	468	488	292	135	37	12

Table S4. Other EJ-related exposure/response modifiers.

Exposure/Response Modifier (ERMs)	Individual or Population Level	Vulnerability or Susceptibility	Biological Consequence (LADD/ADD = C*IR*EF/BW)	Change Tendency to Express Biological Symptoms
Life Stage (Children/Elderly)	I	S	IR, EF, BW	Increase
Sex (Male/Female) [2]	I/P	S	IR, BW	Unknown
Pre-Existing Condition (Asthma, cancer, Diabetes, Respiratory)	I	S	IR, BW	Increase
BMI	I/P	S	BW [3]	Increase
Lack of Education	I	V	BW [4]	Increase
Lack of Health Care	I	V	BW	Increase
Food Deserts	P	V	C, BW	Unknown
Genetic Predisposition	I	S	IR, BW	Increase
Chemical Mixtures	I/P	V	C [5]	Increase
% pre-1960 (Lead Paint)	I/P	V	C, EF [5]	Increase
Proximity NPDES	I/P	V	C, EF [5]	Increase
Proximity NPL Sites	I/P	V	C, EF [5]	Increase
Proximity RMP Facilities	I/P	V	C, EF [5]	Increase
Proximity Traffic	I/P	V	C, EF [5]	Increase
Proximity TSDF	I/P	V	C, EF [5]	Increase
Uses Regulated (Cigarettes, Alcohol) and Unregulated (Drugs) Substances	I/P	V	C, IR	Increase
population density	P	V	C [6]	Increase
% Urban tract	I/P	V	C, EF [6]	Increase

% of Land For Industry, Commerce & Transportation	I/P	V	C, EF [6]	Increase
% Home Owners	P	V	C, EF	Decrease
% of Labor Force in Manufacturing	P	V	IR, EF	Increase
% Immigrated in the 1980s and 1990s	P	S	C, IR, EF, BW	Increase
% of Total Pounds Toxic Releases	I/P	V	C [7]	Increase
% of Pounds of CO, NH_3, No_x, PM, SO_2, VOCs	I/P	V	C [7]	Increase
Low Birth Weight Rate	I/P	S	IR, BW [7]	Increase
Age Distribution	I/P	S	IR, BW [7]	Increase

BMI, Body Mass Index; NPDES, National Pollutant Discharge Elimination System; NPL, National Priorities List; RMP, Risk Management Plan; TSDF, Hazardous waste Treatment, Storage and Disposal Facilities; CO, carbon monoxide; NH_3, Ammonia; NO_x, Nitrogen Oxides; SO_2, Sulfur dioxide; VOCs, Volatile Organic Compound; I, individual; P, Population; V, vulnerability; S, susceptibility; C, concentration; IR, intake rate; BW, body weight; EF, exposure factor.

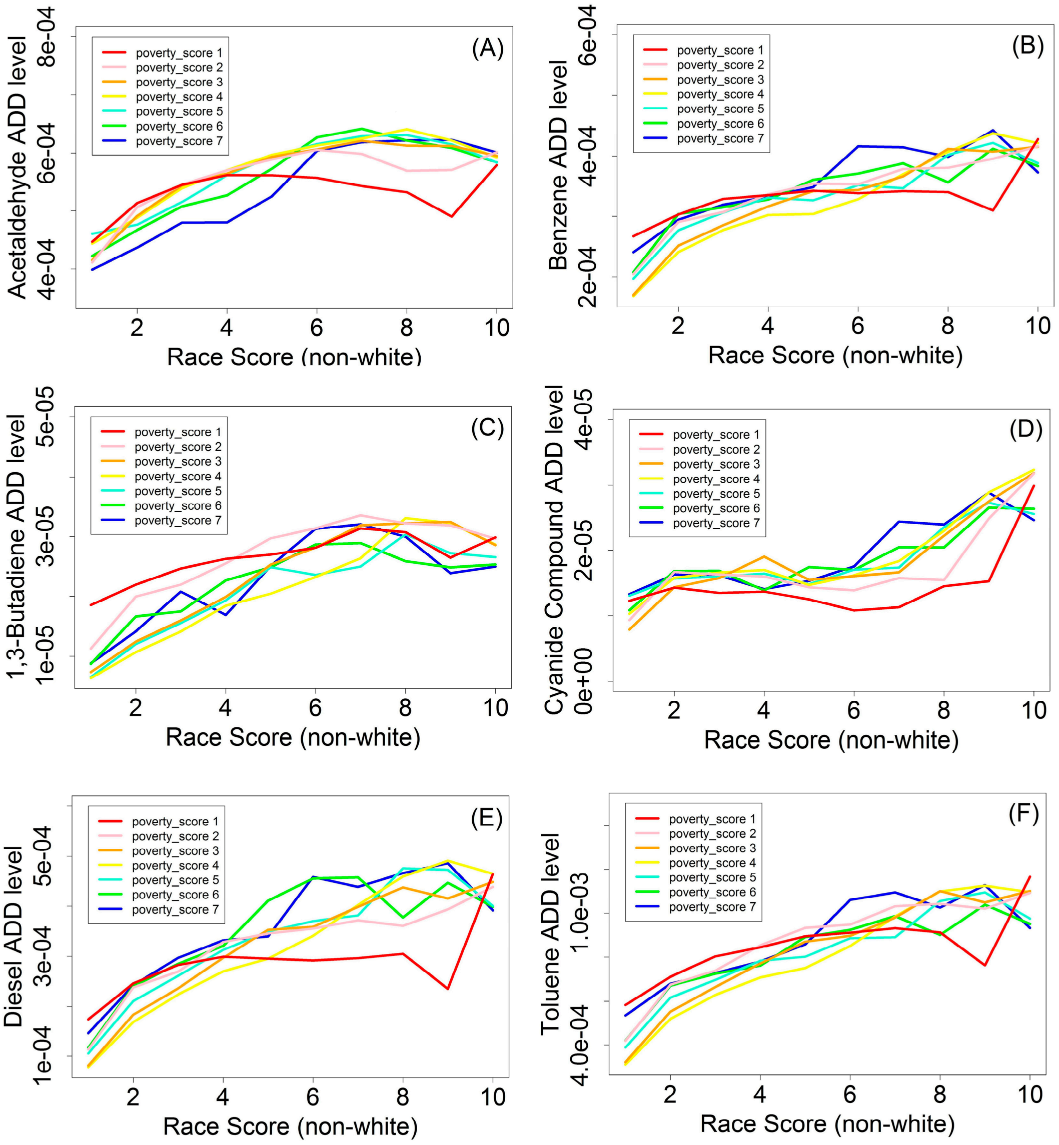

Figure S1. Single-chemical ADD levels (mg-day/kg) associated with both poverty and race scores. (**A**) Acetaldehyde; (**B**) Benzene; (**C**) 1,3-Butadiene; (**D**) Cyanide Compound; (**E**) Diesel PM; (**F**) Toluene.

References

1. U.S. EPA (Environmental Protection Agency), Exposure Factors Handbook. 2011: U.S. EPA, National Center for Environmental Assessment, Washington, DC. EPA/600/R-09/052F. Available: http://cfpub.epa.gov/ncea/risk/recordisplay.cfm?deid=236252 (accessed on 29 December 2015).
2. Clougherty, J.E. A growing role for gender analysis in air pollution epidemiology. *Environ. Health Perspect*. **2010**, *118*, 167–176.
3. Taylor, W.C.; Poston, W.S.C.; Jones, L.; Kraft, M.K. Environmental justice: Obesity, physical activity, and healthy eating. *J. Phys. Act. Health* **2006**, *3*, 30–54.
4. Martin, M.A.; Frisco, M.L.; Nau, C.; Burnett, K. Social stratification and adolescent overweight in the United States: How income and educational resources matter across families and schools. *Soc. Sci. Med*. **2012**, *74*, 597–606.
5. U.S. EPA (Environmental Protection Agency), EJSCREEN Technical Documentation. 2015: U.S. EPA, Office of Policy, Washington, DC, USA. Available online: http://www.epa.gov/sites/production/files/2015-05/documents/ejscreen_technical_document_20150505.pdf (accessed on 21 July 2015).
6. Pastor, M.; Morello-Frosch, R.; Sadd, J.L. The air is always cleaner on the other side: Race, space, and ambient air toxics exposures in California. *J. Urban Aff.* **2005**, *27*, 127–148.
7. Barzyk, T.M.; White, B.M.; Millard, M.; Martin, M.; Perlmutt, L.D.; Harris, F.; Nguyen, P.; Walts, A.; Geller, A. Linking socio-economic status, adverse health outcome, and environmental pollution information to develop a set of environmental justice indicators with three case study applications. *Environ. Justice* **2011**, *4*, 171–177.

© 2016 by the authors; licensee MDPI, Basel, Switzerland. This article is an open access article distributed under the terms and conditions of the Creative Commons by Attribution (CC-BY) license (http://creativecommons.org/licenses/by/4.0/).
